# Roles of Alp and Prt Regulons in the Response of *Pseudomonas aeruginosa* to UV‐C Light

**DOI:** 10.1111/1758-2229.70268

**Published:** 2026-01-12

**Authors:** Marina R. B. Fonseca, Renatta S. Oliveira, Henrique Kustor, Rubia R. L. Freitas, Enzo B. S. Mello, Cristina E. Alvarez‐Martinez, Rodrigo S. Galhardo

**Affiliations:** ^1^ Institute of Biomedical Sciences, Department of Microbiology University of São Paulo São Paulo Brazil; ^2^ Institute of Biology, Department of Genetics, Evolution, Microbiology and Immunology University of Campinas Campinas Brazil

**Keywords:** Alp regulon, DNA damage response, pyocins, SOS regulon, transcriptomics

## Abstract

UV light is a well‐studied environmental DNA damaging agent. Bacterial cells respond to UV exposure by upregulating several pathways to repair and tolerate the lesions induced by this agent. The SOS response is the primary pathway activated during genotoxic stress that can shift the balance between mutagenesis and genome integrity. However, in *Pseudomonas aeruginosa* , the canonical SOS response is not the only pathway activated after DNA damage. This opportunistic pathogen also activates the production of pyocins (Prt regulon) and an autolysis pathway controlling the *alp* genes (Alp regulon) in response to DNA damage. This study aims to characterise gene expression changes in response to UV‐C damage. We performed RNA sequencing analysis to determine the set of differentially expressed genes, and qRT‐PCR to track the course of expression of representative genes from each regulon. Our results show that the canonical LexA‐regulated SOS response is the earliest activated one, while the Alp regulon displays a delayed induction. We also investigated the contribution of the Alp and Prt regulons to UV‐induced cell death and found that the predominant mechanism varies between PAO1 sublines.

## Graphical abstract

Our results show that LexA‐regulated genes are the first to be induced after DNA damage, while autolysis functions controlled by the Alp system are the last. Death by UV is mediated primarily by Alp or Prt systems, depending on the *Pseudomonas aeruginosa* subline.

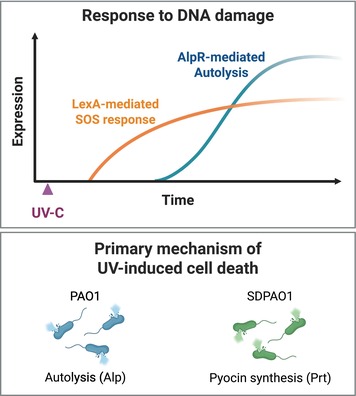

## 1 Introduction

UV light can cause DNA damage, making it a powerful tool for sterilising surfaces. UV light primarily causes two types of DNA lesions, collectively called photoproducts: cyclobutane‐pyrimidine dimers (CPDs) and 6‐4 photoproducts (6‐4PPs) (Goosen and Moolenaar 2008; Douki and Cadet 2001). When life first emerged on Earth, the early atmosphere lacked protective layers capable of filtering UV radiation from the sun. Mechanisms to cope with UV damage were selected along the evolving domains of life, and now they act mainly to protect organisms from UV‐B (295–320 nm) and UV‐A (320–400 nm), since the ozone layer blocks all the UV‐C (< 295 nm), which is the most harmful to living organisms. Nevertheless, UV‐C light is the premier model for DNA damage and repair studies, given its high specificity and high yield of photoproducts, which are also generated by UV‐B and UV‐A (Friedberg et al. 2006).

Upon UV‐induced damage, error‐free mechanisms such as nucleotide excision repair and photoreactivation remove UV‐induced lesions and restore genome integrity (Goosen and Moolenaar 2008; Kisker et al. 2013). Cell division is also temporarily blocked by SulA, an inhibitor of FtsZ polymerisation that prevents cytokinesis until DNA repair is completed (Cordell et al. 2003; Burby and Simmons 2020). FtsZ plays a pivotal role in bacterial cell division, and SulA‐mediated inhibition of its polymerisation has been demonstrated in *Pseudomonas aeruginosa* (Chen et al. 2012).

In parallel, error‐prone translesion synthesis may allow replication across damaged templates, in which case DNA damage tolerance may come at the expense of mutagenesis, potentially granting some individuals in the population an adaptive advantage (Galhardo et al. 2007; Fujii and Fuchs 2020). All these pathways are coordinated by the SOS response, a tightly regulated system triggered when RecA binds to single stranded DNA (ssDNA), forming nucleoprotein filaments that stimulate the autocleavage of the LexA repressor. Under normal conditions, LexA represses the expression of all genes containing an SOS‐box in their promoter sequences (Lima‐Noronha et al. 2022).

*P. aeruginosa* is an opportunistic pathogen that is the leading cause of chronic infections in cystic fibrosis patients and constitutes a global concern regarding antimicrobial resistance (Folkesson et al. 2012). However, it is also found in various environmental niches and, therefore, subject to a range of different stressors, such as UV light (de Sousa et al. 2025).

This bacterium has a unique DNA damage response, composed of three regulons controlling distinct functions, all regulated by repressors of the CI/LexA family that respond to RecA activated by single‐stranded DNA. Although all three regulons are triggered by the same event and controlled by similar repressors, the subsequent cascade of regulatory mechanisms differs. The canonical SOS response, controlled by the LexA repressor, directly regulates 15 genes by binding to 11 SOS operators upstream various genes and operons, according to microarray studies (Cirz et al. 2006).

While in the SOS response the LexA repressor directly controls the expression of each gene in the regulon, in the henceforth called Prt regulon, expression of target genes is induced by a transcriptional activator (PrtN), normally repressed by the LexA‐like repressor PrtR (Matsui et al. 1993). The Prt regulon consists of 43 genes involved in the production and release of pyocins, including genes specifically involved in cell lysis (Michel‐Briand and Baysse 2002; Turnbull et al. 2016). Similarly, the induction of a third regulon (hereafter referred to as the Alp regulon), containing the *alp* genes, requires an antiterminator (AlpA), normally repressed by the regulator AlpR. AlpA positively regulates six genes in another autolysis pathway and a second group of genes on the positive strand between PA0807 and PA0829 with unknown function (McFarland et al. 2015; Peña et al. 2021).

The DNA damage response of *P. aeruginosa* is therefore unique, in the sense that seemingly antagonistic functions are induced. While the SOS response favours cellular maintenance pathways, activation of Prt and Alp regulons ultimately leads to cell death, in a process where individual cells sacrifice themselves for the benefit of the population. Indeed, several studies have shown that pyocin production induced by DNA damage sensitises *P. aeruginosa* to DNA‐damaging agents such as UV light and the widely used antimicrobial ciprofloxacin (Penterman et al. 2014; Sun et al. 2014; Fan et al. 2019).

To further understand this intricate response, we conducted a comparative transcriptomic analysis between UV‐treated *P. aeruginosa* PAO1 cells and untreated controls to investigate the global transcriptional changes after DNA damage. We also carried out a detailed time‐course expression analysis of representative genes from the three regulons to establish their induction kinetics, and a genetic analysis to assess how the Alp and Prt regulons contribute to UV‐induced cell death. Therefore, we aimed to define the transcriptional and physiological responses of *P. aeruginosa* to UV‐C light and to determine how the Alp and Prt regulons contribute to cell death and survival outcomes.

## 2 Material and Methods

### 2.1 Bacterial Strains and Growth Conditions

Bacterial strains and plasmids are listed in Table 1. The PAO1 strain used in our lab (referred to as PAO1 throughout this manuscript) was generously supplied by Dr. Regina Baldini (Institute of Chemistry, University of São Paulo). We constructed a set of isogenic strains with deletions in *alpA* and *prtN* using the plasmids pEXG2‐ΔalpA and pEXG2‐ΔprtN (Table 1) through a two‐step selection process (Hmelo et al. 2015). Another set of strains was kindly provided by Dr. Simon Dove (Boston Children's Hospital); these were generated using the same deletion constructs, with the parental strain referred to as SDPAO1 throughout this manuscript.

**TABLE 1:** Bacterial strains and plasmids used in this study.

Bacteria	
Strain	Source
PAO1	Regina Baldini, University of Sao Paulo
PAO1 Δ*alpA*	This study. Constructed using pKMC1057 (pEXG2‐ ∆*alpA*) and PAO1 parental strain
PAO1 Δ*prtN*	This study. Constructed using pKMC1059 (pEXG2‐∆*prtN*) and PAO1 parental strain
PAO1 Δ*alpA* Δ*prtN*	This study. Constructed using pKMC1059 (pEXG2‐∆*prtN*) and PAO1 Δ*alpA* parental strain
PAO1 attTn7::P*recA–luxCDBAE*	Valencia et al. (2017)
PAO1 P*imuA::mTurquoise2*	This study. Constructed using mini‐CTX2‐P*imuA*::*mTurquoise2* and PAO1 parental strain
PAO1 P*alpB*::*lacZ*	This study. Constructed using pJP01 (mini‐CTX‐P*alpB*‐*lacZ*) and PAO1 parental strain
PAO1 Δ*alpA* P*alpB*::*lacZ*	This study. Constructed using pJP01 (mini‐CTX‐P*alpB*‐*lacZ*) and PAO1 Δ*alpA* parental strain
SDPAO1	Simon Dove, Boston Children's Hospital
SDPAO1 Δ*alpA*	McFarland et al. (2015)
SDPAO1RGΔ*prtN*	McFarland et al. (2015)
SDPAO1RGΔ*alpA/*Δ*prtN*	This study. Constructed using pKMC1059 (pEXG2‐∆*prtN*) and SDPAO1 Δ*alpA* parental strain
SDPAO1 P*alpB*::*lacZ*	This study. Constructed using pJP01 (mini‐CTX‐P*alpB*‐*lacZ*) and SDPAO1 parental strain
SDPAO1 Δ*alpA* P*alpB*::*lacZ*	This study. Constructed using pJP01 (mini‐CTX‐P*alpB*‐*lacZ*) and SDPAO1 Δ*alpA* parental strain
Plasmids	
mini‐CTX‐P*alpB*‐*lacZ*	(Peña et al. (2021))
mini‐CTX2‐P*imuA*::*mTurquoise2*	This study. Constructed as described in Materials and Methods

### 2.2 Growth and UV Irradiation Conditions

UV irradiation was performed by exposing cells to a Philips TUV 15 W/G15T8 lamp (emission peak at 254 nm). For UV irradiation experiments, a saturated culture of the PAO1 strain was diluted to an optical density (OD_600_) of 0.1 in Mueller–Hinton medium and cultivated at 37°C with shaking at 200 rpm until mid‐log phase (OD_600_ 0.4–0.5), at which point a first control sample was collected and the culture was exposed to UV‐C light. We selected this growth stage because mid‐log phase cells exhibit high metabolic activity and active DNA replication, conditions under which DNA repair and stress‐response pathways are more important for survival to UV‐induced damage.

For expression profiles, broth cultures were exposed to 45 J/m^2^ and returned to the incubator at 37°C. Samples were collected at various time points after irradiation, depending on the subsequent experiment. RNA extraction for qRT‐PCR analysis was performed using samples collected over up to 3 h, while samples for total RNA sequencing were collected 60 min after exposure. In all experiments, cultures were kept in the dark after UV‐C treatment to prevent photoreactivation.

Survival experiments with planktonic cultures were performed with cells grown in Mueller–Hinton and irradiated as described above. Immediately after UV irradiation, cultures were serially diluted and plated for colony forming units (CFU) count determination. Additionally, survival experiments were performed with spot tests, in which cells were serially diluted and plated on Mueller–Hinton agar. The plates were then irradiated with different UV doses and photographed after 18–20 h of incubation at 37°C.

### 2.3 Promoter Activity Quantification

The activity of the *recA* promoter was measured using a luciferase reporter in the strain PAO1 attTn7::P*recA*‐*luxCDBAE* (Valencia et al. 2017). Luminescence was measured in technical triplicates using the GloMax Multi+Detection System (Promega, USA) with 96‐well plates. Additionally, OD_600_ was measured to normalise luminescence by cell density.

Integrative plasmid mini‐CTX‐P*alpB*‐*lacZ* (Peña et al. 2021), which contains the *alpB* promoter region (−115 to +117) with its early termination site fused to the *lacZ* reporter, was transformed into the PAO1 and SDPAO1 strains, and recombinant colonies were selected with tetracycline (35 μg/mL). Overnight cultures in Mueller–Hinton medium were diluted to an OD_600_ of 0.1 and incubated at 37°C to mid‐log phase (OD_600_ 0.4–0.5). At this point, β‐galactosidase activity in an untreated sample was measured, and cultures were irradiated with UV‐C light and incubated in the dark to prevent photoreactivation. β‐galactosidase activity was measured again at 15, 30, 45, 60 and 120 min after treatment. For the β‐galactosidase assay, cells were lysed with chloroform and SDS, and o‐nitrophenyl‐β‐D‐galactoside (ONPG) was subsequently added. Reactions were incubated for 5 min at 28°C before the addition of Na_2_CO_3_ stop solution. Absorbance was measured at 420 nm and Miller units were calculated (Miller 1972) by normalising against the optical density of the culture (OD_600_).

To quantify the promoter activity of a LexA‐regulated gene, we designed a pBBRMCS‐2 vector containing the promoter region (−350 to +18) of the *imuA* gene (PA0671) fused with mTurquoise2 fluorescent protein. The vector was constructed by sequence and ligase independent cloning (SLIC) by amplifying target sequences with oligos described in Supplementary File S1 and digesting the vector with *SmaI* enzyme. Next, we used *BamHI* and *HindIII* enzymes to release the region of interest P*imuA*::*mTurquoise2* and cloned this fragment with T4 DNA ligase into the mini‐CTX2 vector, which was also digested with the same enzymes. Recombinant colonies were selected with tetracycline (35 μg/mL). The mini‐CTX2 vector containing P*imuA*::*mTurquoise2* was transferred to *P. aeruginosa* PAO1 to obtain a chromosomally located single‐copy reporter. To assay P*imuA* activity, cultures were grown overnight and then diluted in Mueller–Hinton medium to an OD_600_ of 0.1 and incubated with agitation at 37°C until reaching mid‐log phase. Control aliquots were collected, and the cultures were irradiated with 45 J/m^2^ of UV‐C light. Samples were collected every 15 min after irradiation for up to 1 h. For efficient fluorescence detection, the samples were centrifuged for 2 min at 7000 rpm and resuspended in an equivalent volume of saline. Measurements were performed in at least two technical replicates of 200 μL in a 96‐well Greiner Bio‐One black plate with a clear bottom in SpectraMax Paradigm plate reader. Experiments were performed in triplicate. Fluorescence detection was performed with excitation at 434 nm and detection at 474 nm, wavelengths corresponding to the excitation and emission peaks of the mTurquoise2 protein (https://www.fpbase.org). The fluorescence was normalised against the OD_600_ of each sample.

### 2.4 Total RNA Extraction and Removal of Ribosomal RNA

Total RNA was extracted from each sample using RNeasy Protect Bacteria Reagent (Qiagen), according to the manufacturer's instructions. RNA concentration was determined by measuring the OD_260_, and RNA integrity was verified using the Agilent 2100 Bioanalyzer (Agilent Technologies, Santa Clara, CA, USA). Samples with an RIN ≥ 7.5 were considered suitable for downstream analysis and were maintained at −80°C. The Ribo‐Zero rRNA Removal Kit (Illumina, San Diego, CA, USA) was used to remove rRNA according to the manufacturer's instructions. The rRNA depletion was verified using an Agilent 2100 Bioanalyzer (Agilent Technologies, Santa Clara, CA, USA).

### 2.5 RNA Sequencing and Bioinformatics Analyses

The TruSeq RNA Sample Prep Kit (Illumina, San Diego, CA, USA) was used to construct the libraries. The cDNA libraries were normalised to 4 nM prior to cluster generation and sequenced using a NextSeq 500/550 Mid Output cartridge (Illumina, San Diego, CA, USA). The quality of raw reads was verified using FastQC (Babraham Bioinformatics). Bioinformatic analyses were performed by quality‐filtering reads with BBDuk (BBTools by Brian Bushnell), mapping reads to the reference genome with BWA (Li and Durbin 2009) and calculating differential expression with DESeq2 (Love et al. 2014) in Geneious Prime (Biomatters).

### 2.6 Quantitative RT‐PCR

Quantitative real‐time PCR experiments were performed on three independent biological samples, each in triplicate, using *rpsL* (30S ribosomal protein S12) as a reference control, due to its stable expression under the experimental conditions. The primers used are listed in Supplementary File S1. Reactions were performed using Power SYBR Green PCR master mix (Applied Biosystems, Foster City, CA, USA). Primers were designed using the software Primer3 (http://frodo.wi.mit.edu/). Total RNA was extracted using TRIzol reagent (Invitrogen Life Technologies, Carlsbad, CA, USA), following the manufacturer's instructions. RNA integrity was analysed by agarose gel electrophoresis, and the concentration was determined by measuring the OD_280_. RNA samples were treated with deoxyribonuclease I (Promega, Madison, WI, USA) and tested for the absence of genomic DNA contamination by PCR. cDNA synthesis was carried out in 20 μL reactions using the High‐Capacity cDNA Reverse Transcription Kit (Applied Biosystems, Foster City, CA, USA). Real‐time PCR reactions were carried out in a final volume of 12 μL containing 6 μL Power SYBR Green PCR master mix, 0.5 μL of each primer (10 μM), 50 ng/μL cDNA and 4 μL ultra‐pure water. The reaction conditions were 95°C for 10 min, followed by 40 cycles comprising 95°C for 15 s and 60°C for 1 min. Dissociation curves were generated at the end of each PCR cycle to verify the amplification of a single product. Relative expression was quantified by calculating gene level changes, adjusting for primer amplification differences (Pfaffl 2001).

## 3 Results

**FIGURE 1:**
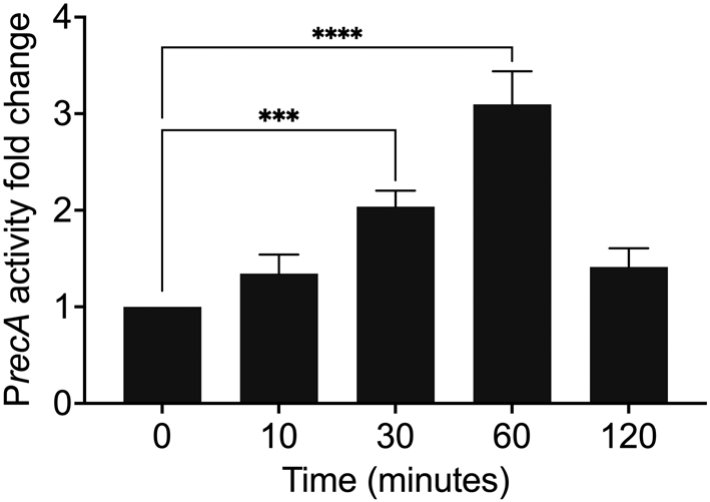
*recA* promoter activity in response to UV‐C light irradiation. Luminescence from PAO1 attTn7::P*recA*–*luxCDBAE* reporter was measured at 0, 10, 30, 60 and 120 min after exposure to 45 J/m^2^ of UV‐C and normalised to OD_600_. Bars represent mean ± SD of *n* = 3 independent biological replicates (each in technical triplicates). Differences over time were assessed by ordinary one‐way ANOVA with Dunnett's correction for multiple comparisons to 0 min (GraphPad Prism). Asterisks denote two‐tailed, multiplicity‐adjusted *p* values: *p < 0.05, **p < 0.01, ***p < 0.001, ****p < 0.0001.

Initially, we assessed the survival of PAO1 under increasing doses of UV‐C light. Given our goal of analysing gene expression, we avoided temporarily holding the cells in saline or PBS during UV exposure, as these conditions may themselves induce changes in gene expression. Therefore, we chose to use Mueller–Hinton medium instead of the more commonly used LB medium, since the UV‐C light (260 nm) transmittance of Mueller–Hinton is 20%, whereas that of LB is only 0.1%, as measured with a spectrophotometer under our laboratory conditions. Exposure of mid‐log‐phase cultures to 45 J/m^2^ of UV‐C resulted in an average survival rate ranging from 10% to 50%. Because *recA* is a key gene of the canonical SOS regulon controlled by LexA, we next monitored its promoter activity using a P*recA*::*luxCDBAE* reporter under the same conditions to identify the peak of induction. The activity of the P*recA* reporter reached its maximum relative increase of ~3‐fold at 60 min after treatment with 45 J/m^2^ of UV‐C (Figure 1).

### 3.1 Transcriptome Changes Induced by UV‐C

We performed transcriptomic analysis of PAO1 irradiated with 45 J/m^2^ and allowed 1 h of recovery before RNA extraction. UV‐C irradiation led to differential expression (≥ 2‐fold) of 96 genes (Table 2). As reported in Table 2, among the 94 upregulated genes, 51 genes were induced more than fivefold. All genes that were at least twofold differentially regulated after UV‐C irradiation are listed in the Supplementary File S1.

**TABLE 2:** Effects of treatment with UV‐C light on mRNA levels.

Change in mRNA level	Number of genes			
	Total	≥ 2–5‐fold	≥ 5–10‐fold	≥ 10–20‐fold
Increase	94	43	26	25
Decrease	2	2		
Total	96	45	26	25

The three regulons combined control the expression of 86 genes (Matsui et al. 1993; Michel‐Briand and Baysse 2002; Cirz et al. 2006; McFarland et al. 2015; Peña et al. 2021), 76 of which were differentially regulated in our analysis (Supplementary File S1). Out of the 15 known SOS genes, only two, PA1044 and PA1045, did not show altered expression in our analysis. PA1044 encodes a hypothetical protein, and PA1045 encodes the DNA helicase DinG. Although both genes are directly regulated by LexA, their differential expression in ciprofloxacin microarray studies was modest, consistent with our results (Blázquez et al. 2006; Cirz et al. 2006). Therefore, our transcriptomic analysis revealed 18 upregulated genes and two downregulated genes that are not directly known to be regulated by LexA, PrtR/PrtN or AlpR/AlpA, according to previously published data. Our results confirm the major roles of LexA‐, PrtR‐ and AlpR‐regulated genes in the DNA damage response of *P. aeruginosa* .

### 3.2 Time Course of Induction of Genotoxic Stress Responses

**FIGURE 2:**
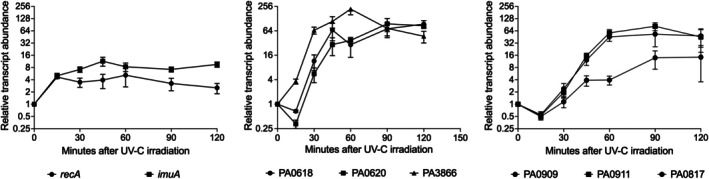
Time course expression of genes belonging to LexA, Prt and Alp regulons after UV‐C damage. Real‐time PCR of representative genes from each group showing the kinetics of activation for each regulon. A—The LexA‐regulated SOS response was measured by the relative transcript abundance changes over time of genes *recA* and *imuA*. B—The Prt system was measured by the relative transcript abundance changes over time of genes PA0618, PA0620 and PA3866. C—The Alp system was measured by the relative transcript abundance changes over time of genes *alpC* (PA0909), *alpE* (PA0911) and PA0817. Values and error bars reflect mean ± SD of *n* = 3 biological replicates in technical triplicates.

Since our results confirmed that genes related to the Prt and Alp regulons are strongly upregulated by UV‐C, we conducted a gene expression analysis to understand the timing of induction of the different regulons. Figure 2 shows the qRT‐PCR results illustrating the time course of the activation of randomly selected genes from each of the three DNA damage‐responsive regulons. To assess the expression kinetics of the LexA regulon, we analysed the *recA* and *imuA* genes, which encode the recombination protein and SOS regulator RecA and a protein that participates in a damage‐inducible mutagenesis pathway, respectively (Galhardo et al. 2005; Warner et al. 2010). The PrtN regulon was interrogated by analysis of the expression of PA0618 and PA0620, two pyocin R2 genes that are arranged in one operon, as well as PA3866, which encodes pyocin S4 (Matsui et al. 1993; Michel‐Briand and Baysse 2002; Penterman et al. 2014). The Alp system expression was evaluated by analysing the transcriptional profiles of *alpC* (PA0909) and *alpE* (PA0911) genes, which are organised in the same operon encoding the lysis cassette, as well as PA0817, a gene from a small cluster of AlpA‐regulated genes of unknown function (McFarland et al. 2015; Peña et al. 2021).

The SOS response is the fastest activated one, as early as 15 min after UV damage (Figure 2). We observed an apparent decrease in the expression of AlpA‐regulated genes and pyocin R2 genes in the early stages of the DNA damage response. PrtN‐regulated genes exhibited heterogeneity; for instance, PA3866 was activated early, similarly to LexA‐regulated genes, and in contrast to pyocin R2 genes. By 30 min after UV irradiation, the Alp regulon and pyocin R2 genes were increasing their expression, while the SOS response is already near its peak. These results show that although UV triggers the induction of all three regulons, there is considerable variation in the timing of each one's activation.

**FIGURE 3:**
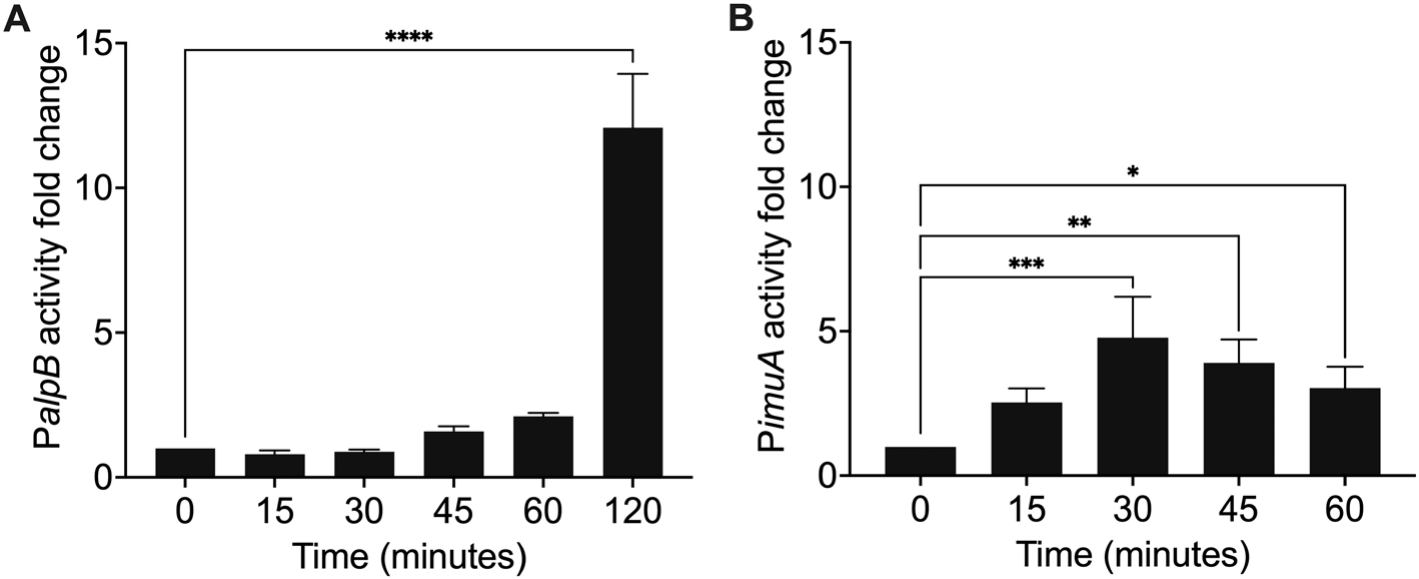
Promoter activities of *alpB* and *imuA* after UV‐C. (A) β‐galactosidase activity (Miller units) from PAO1 carrying mini‐CTX‐P*alpB*‐lacZ measured at 0, 15, 30, 45, 60 and 120 min after exposure to 45 J/m^2^ UV‐C. (B) Fluorescence normalised to OD_600_ from PAO1 carrying mini‐CTX‐P*imuA*::*MTurquoise2* measured at 0, 15, 30, 45 and 60 min after exposure to 45 J/m^2^ UV‐C. Bars represent mean ± SD of *n* = 3 independent biological replicates (technical duplicates). Differences over time were assessed by ordinary one‐way ANOVA with Dunnett's correction for multiple comparisons to 0 min (GraphPad Prism). Asterisks denote two‐tailed, multiplicity‐adjusted *p* values: *p < 0.05, **p < 0.01, ***p < 0.001, ****p < 0.0001.

To confirm the results obtained by qRT‐PCR, we performed reporter assays with the promoters of *alpB* (Figure 3A) and *imuA* (Figure 3B). Figure 3A shows the fold change in expression after irradiation compared to the unirradiated sample. The results are consistent with our qRT‐PCR data, showing that the expression of the *alpB* reporter system declines slightly after 15 min and increases above basal levels between 30 and 45 min. The experiment was extended to ensure the detection of the maximum activity, revealing that expression increases more than 13‐fold 2 h after exposure to UV‐C. Immediately after UV‐C exposure, β‐galactosidase assays also indicate a slight decrease in *alpB* expression, although this difference is not statistically significant.

Figure 3B shows the increase in *imuA* promoter activity by detecting fluorescence in cultures (normalised by OD_600_) after exposure to UV‐C light. As seen in the qRT‐PCR results, the *imuA* expression rapidly increased 2.5‐fold within 15 min after exposure. qRT‐PCR measures the expression after transcription, whereas reporter assays depend on transcription, translation and protein maturation for signal detection; therefore, a delay in reporter response is expected in this case. Despite this limitation, the data clearly confirm the earlier induction of LexA‐regulated genes in contrast to the late induction of AlpA‐regulated genes. These results show that the three regulons are all induced after UV exposure, but with distinct timing: the SOS response is activated first, followed by delayed induction of the Prt and Alp regulons.

### 3.3 Contribution of Alp and Prt Regulons to Death by UV‐C

Previous studies have shown that both the PrtN and AlpA regulons promote DNA damage‐induced cell death (Penterman et al. 2014; Sun et al. 2014; Fan et al. 2019). Pyocin production driven by the PrtN regulon appears to be the major factor involved, given that strains carrying an uncleavable *prtR* allele are much more resistant to UV light than the wild‐type strain (Penterman et al. 2014). Nevertheless, the role of AlpA‐regulated genes in UV resistance has not yet been investigated in detail, and direct comparisons between the contributions of the Prt and Alp regulons have not been performed. Here we expand on these earlier findings by systematically analysing both regulons under identical experimental conditions, revealing distinct patterns of UV‐C induced death among different *P. aeruginosa* backgrounds.

Beyond its role in controlling pyocin production, PrtR also regulates additional pathways, including genes of the Type III secretion system (Jiao et al. 2021). This broader regulatory function suggests that the PrtR–PrtN axis may integrate multiple stress responses, further emphasising the need to dissect its contribution to UV‐induced cell death. Based on these findings and our observation that the Alp regulon is activated later, we compared UV resistance in strains lacking Alp and Prt activators to the parental strain.

**FIGURE 4:**
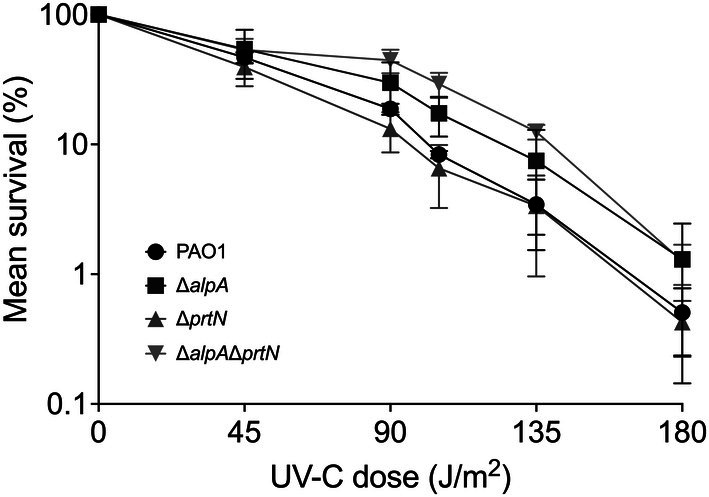
Survival of PAO1 and its derivatives to UV‐C irradiation. The results represent the average of three independent experiments for each strain, and the error bars represent the standard error. Statistical analysis was performed using Student's *t* test, by comparing survival of the mutant strains with the WT strain at each UV‐C dose. Significant differences (*p* < 0.05) were observed for 45, 90 and 135 J/m^2^ when comparing the Δ*alpA* Δ*prtN* double mutant with the WT strain. The remaining comparisons are not significant (*p* > 0.05).

First, we performed experiments in which all strains were irradiated as commonly used in experiments in the photobiology field: liquid cultures were exposed to increasing UV doses, then immediately diluted and plated for CFU count determination. With this method, we observed no effect of deleting *prtN* or *alpA* alone, while deleting both *alpA* and *prtN* conferred a minor protection against some UV doses (Figure 4).

**FIGURE 5:**
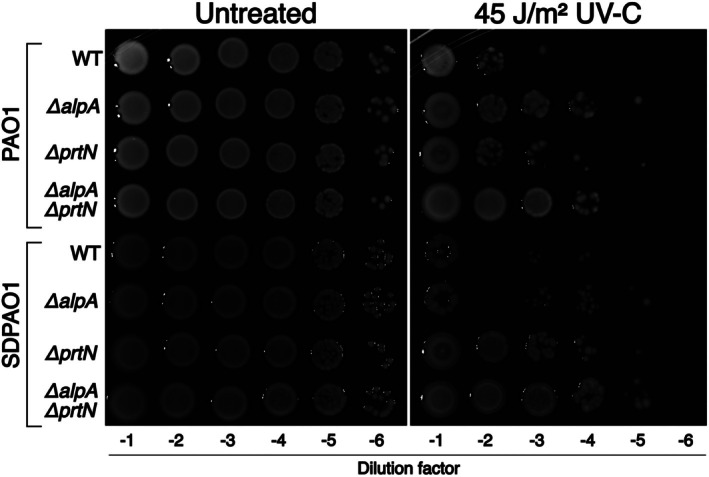
Growth of different PAO1 and SDPAO1 strains and their derivatives after UV irradiation. Serial dilutions of cultures adjusted to OD600 0.5 were spotted on Mueller–Hinton agar plates. Plates were irradiated or not with UV‐C light as indicated and subsequently incubated in the dark at 37°C for 24 h before photographs were taken.

Next, we attempted to verify the marked UV resistance of pyocin production‐deficient strains reported in the literature, using the same type of experiment described in that report (Penterman et al. 2014). We evaluated the survival of cells previously diluted and spotted on agar plates and then UV‐irradiated. In these qualitative experiments, pyocin production likely exerts a stronger local effect because cell density remains high in dilution spots after UV irradiation. The results indicate a major role of Alp‐regulated genes in cell death. PAO1 Δ*alpA* strain was significantly more resistant to UV‐C light than the wild‐type counterpart. Deletion of *prtN* had a minor effect on survival, and the double mutant was as resistant as the Δ*alpA* strain (Figure 5). This finding contrasts sharply with the previous reports showing a major role of Prt‐regulated pyocin R2 production in cell death following DNA damage.

Given the known variability between PAO1 sublines (Klockgether et al. 2010; Sidorenko et al. 2017), we investigated whether the absence of a deleterious effect of pyocin production on UV‐C survival could be due to the strain's genetic background. We obtained the same isogenic set of strains constructed in the SDPAO1 background and tested them for UV‐C survival. In this case, *prtN* deletion had the greatest impact on UV resistance, substantially improving survival. Given that most experiments previously reported in the literature were performed with cells grown and plated on LB medium, we repeated these experiments with liquid bacterial cultures under the same conditions (Figure S1). Even with this experimental setup, the results clearly show that the Alp regulon is the most significant contributor to cell death following UV‐C exposure in our PAO1 strain. In LB medium, the results obtained with the SDPAO1 set of strains once again reproduced the findings of the literature: absence of pyocin production due to *prtN* deletion rendered cells UV‐C‐resistant, and the double mutant lacking both *prtN and alpA* is even more resistant to UV. In both culture media, we observed the same phenomenon of density‐dependent killing previously reported, where cell death is also influenced by local cell density, likely due to pyocin production by neighbouring siblings that kill cells transiently sensitive to pyocins (Penterman et al. 2014). This phenomenon can be observed as an apparent lack of consistency in serial dilutions and was reproducible (Figures 5 and S1).

**FIGURE 6:**
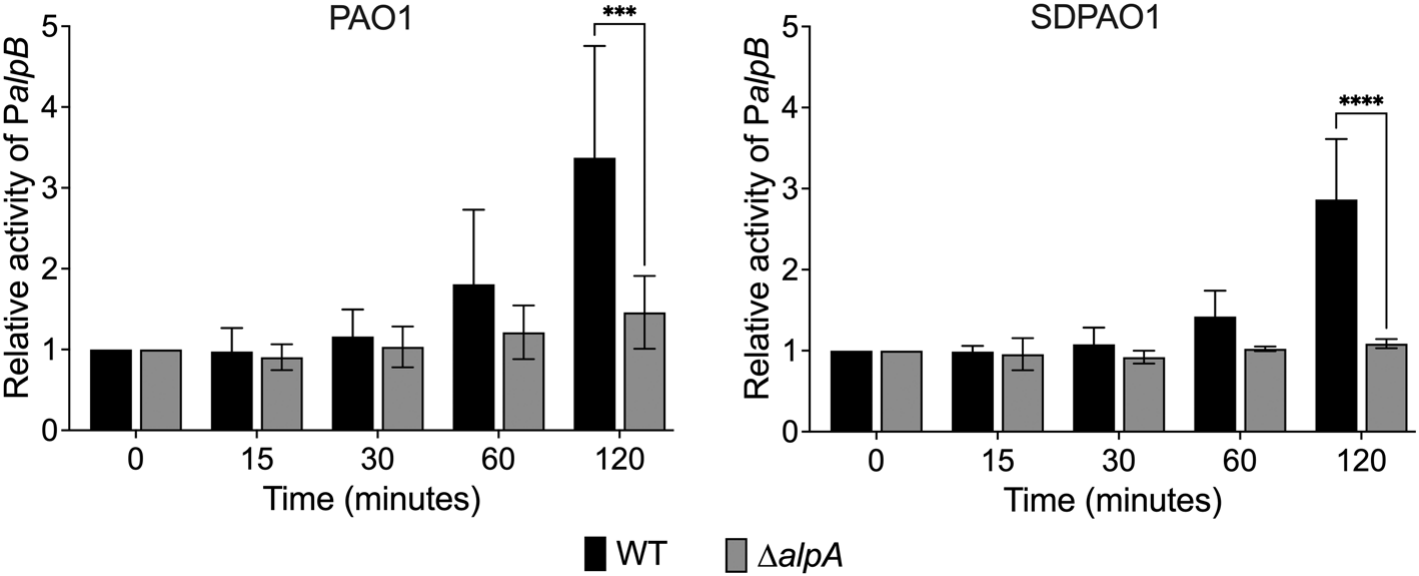
Activity of P*alpB* in PAO1 and SDPAO1 backgrounds. Promoter activity from a P*alpB* reporter was measured at 0, 15, 30, 60 and 120 min after exposure to 10 J/m^2^ UV‐C in PAO1 and SDPAO1 backgrounds, comparing WT and Δ*alpA*. Bars represent mean ± SD of *n* = 3 independent biological replicates (each in technical duplicates). Statistics: For each background, data were analysed by ordinary two‐way ANOVA (factors Genotype and Time, including their interaction), followed by Šidák‐adjusted pairwise comparisons of WT versus Δ*alpA* at each time point; two‐tailed, multiplicity‐adjusted *p* values (GraphPad Prism). Asterisks denote adjusted significance levels: *p < 0.05, **p < 0.01, ***p < 0.001, ****p < 0.0001.

We next asked whether the difference in the role of the Alp regulon between PAO1 and SDPAO1 could be due to differences in the timing of expression *of alp* genes, as reported in Figure 2. For this purpose, we determined the kinetics of *alpB* reporter expression in wild‐type and *alpA* mutants of both backgrounds (Figure 6). We used a lower UV‐C dose (10 J/m^2^) to minimise the effects of cell death following UV irradiation. The kinetics of *alpB* induction was identical in both parental strains and equally abolished in both *alpA* mutants, indicating that the sharp difference in the role of *alpA* in survival between SDPAO1 and PAO1 is not due to differences in the timing of *alp* genes expression. These findings demonstrate that the effect of Alp‐ and Prt‐regulated genes on UV‐C survival varies depending on the strain background.

## 4 Discussion

In this study, we determined the set of genes differentially expressed in response to DNA damage caused by UV light, a major environmental genotoxin used both in DNA repair studies and in practical applications for surface decontamination in various settings. We used expression of the canonical SOS response (LexA‐regulated) as a proxy to determine 60 min as the optimal time point to investigate changes in gene expression following UV‐C damage. When comparing our UV‐C results to previously published ciprofloxacin microarray data, the most striking difference we observed was in the downregulated transcripts. While 408 genes showed at least a 2‐fold decrease in expression 2 h after ciprofloxacin addition, only two genes were downregulated in our experiments (Cirz et al. 2006). A more recent study examined the transcriptome changes induced by UV‐C light in *P. aeruginosa* , immediately after irradiation, with no recovery period allowed, resulting in 472 upregulated and 214 downregulated genes (Li et al. 2021). Most strikingly, among the three known damage‐inducible regulons, only LexA‐regulated genes showed altered expression in that study, corroborating our finding that LexA‐regulated genes are expressed before Prt and Alp regulons. Several factors may contribute to the observed differences, including differences in PAO1 strains used, time points selected for analysis, and growth conditions.

Also, in agreement with our results, two transcriptomic analyses of *P. aeruginosa* exposed to hydrogen peroxide were conducted at the early stages of the oxidative stress, specifically after 10 and 20 min of exposure. Pyocin genes were altered by two‐ to fivefold only at the latter time point (Palma et al. 2004; Chang et al. 2005). SOS genes were found at higher expression levels in both time points, with fold‐induction rates from two‐ to 11‐fold. Our results obtained with UV‐C also reveal the same pattern of earlier induction of the LexA‐regulated genes compared to at least some of the PrtR‐regulated genes. Similarly, in the transcriptomic analysis data obtained after ciprofloxacin exposure (Cirz et al. 2006), samples were collected at two points: 30 and 120 min after exposure. Although only the 120‐min exposure profile was used to define the SOS response, the available 30‐min data show that, consistent with our observations, most SOS genes showed slight alterations, unlike the Alp and Prt‐regulated genes. No oxidative stress‐associated genes were differentially expressed in our dataset. In a previous study reporting global transcriptomic analysis of the response to hydrogen peroxide‐induced oxidative stress in *P. aeruginosa* , SOS and Pyocin genes were induced, except for PA1151 gene, a pyocin S2 immunity protein, which was repressed (Chang et al. 2005). In our study, PA1151 was induced more than threefold in response to UV‐C irradiation.

We identified 18 genes that were induced by UV‐C and are not known to be regulated by any LexA‐like repressor (Supplementary File S1). Only two of these genes, PA5470 and PA5471 (*armZ*), were also induced in ciprofloxacin and hydrogen peroxide microarray analyses (Palma et al. 2004; Cirz et al. 2006). The *armZ* gene encodes a transcriptional regulator that can activate and repress the expression of several virulence factors in *P. aeruginosa* (Baynham et al. 2006; Pryor et al. 2012). In the gene expression analysis conducted immediately after UV‐C irradiation (Li et al. 2021), several of these 18 genes that are not part of the three known damage‐responsive regulons also exhibited differential expression: PA1466, PA1865, PA1866, PA2284, PA2285 and PA2287. These results confirm that there are additional layers of regulation in the DNA damage response of *P. aeruginosa* that are yet to be elucidated. Interestingly, PA1865, a gene associated with nuclease activity, was induced both by UV‐C light and ciprofloxacin but was repressed by hydrogen peroxide treatment. This pattern suggests a damage‐type‐specific regulation. PA1865 has a conserved role in guiding the unhooking of DNA interstrand cross‐links, functioning as a DNA repair gene (Gwon et al. 2014; Jin et al. 2018).

The heterogeneity in the timing of gene expression within the PrtN regulon is surprising, but it is interesting to observe that pyocin R2 production is a major source of cell death under DNA damaging conditions (Brazas and Hancock 2005; Penterman et al. 2014). Therefore, delaying its production, as observed for genes PA018 and PA0620, may be a strategy that allows cells time to circumvent DNA damage before resorting to the extreme measure of cell lysis to release pyocins. The Prt system is activated when PrtR autocleavage derepresses the *prtN* gene, which encodes a transcriptional activator that stimulates the expression of downstream genes in the regulon (Matsui et al. 1993). The Alp system is controlled by a similar repressor, AlpR, which regulates the *alpA* gene, which in turn encodes an antiterminator that binds to the RNA polymerase, enabling it to bypass intrinsic terminators located downstream (Peña et al. 2021). Our data indicate that although the three regulons share a common inducing signal, differences in the subsequent regulatory events may play an important role in the timing of the response. Additionally, AlpR, PrtR and LexA may also exhibit differences in autocleavage kinetics depending on their affinity for the DNA sequence they interact with, as well as their affinity for RecA nucleofilaments, potentially explaining the results observed in Figure 2.

The roles of AlpA‐ and PrtN‐regulated genes in sensitising *P. aeruginosa* to UV‐C are remarkably variable. We observed that in our PAO1 strain background, previously used in DNA repair and mutagenesis studies (Valencia et al. 2017), autolysis mediated by AlpA‐regulated genes is the main source of cell death following UV exposure. In contrast, in the SDPAO1 background, PrtN‐regulated genes contribute more significantly to UV‐induced cell death. Future comparative genomics and transcriptomics of both strain backgrounds will be essential to identify the genetic differences responsible for this variation. The effect of pyocin production appears to result from a downregulation of LPS biosynthesis genes induced by UV (Penterman et al. 2014). In the model proposed by Penterman et al. (2014), some cells in the population become activated for pyocin production, while the remaining population is sensitised to pyocins released by their siblings due to this decrease in LPS synthesis. Notably, none of the LPS biosynthesis genes previously associated with pyocin resistance were repressed in our transcriptomic analysis (Supplementary File S1), which may partially explain the minor role of PrtN‐dependent cell death under our experimental conditions.

Overall, our results showed that the three damage‐inducible responses of *P. aeruginosa* are activated at distinct times after UV‐C exposure. This temporal aspect of the DNA damage response is consistent with observations made using other DNA‐damaging agents, although such comparisons had not been systematically analysed until now. Our analysis also revealed additional genes regulated by DNA damage, whose regulatory mechanisms remain to be discovered.

Together, these findings reveal a coordinated, multiregulon response in *P. aeruginosa* that balances immediate DNA repair with delayed processes like cell lysis and pyocin release. The distinct timing of regulon activation, coupled with strain‐specific differences in UV resistance, reveals the complexity of bacterial adaptation to genotoxic stress, highlighting the intricate regulatory mechanisms that control the balance between survival and programmed cell death in *P. aeruginosa* .

## Author Contributions

**Marina R. B. Fonseca:** conceptualization, data curation, investigation, formal analysis, writing – review and editing, writing – original draft. **Renatta S. Oliveira:** investigation, formal analysis, writing – review and editing. **Henrique Kustor:** writing – review and editing, investigation, formal analysis. **Rubia R. L. Freitas:** investigation, formal analysis, writing – review and editing. **Enzo B. S. Mello:** investigation, formal analysis, writing – review and editing. **Cristina E. Alvarez‐Martinez:** supervision, formal analysis, writing – review and editing. **Rodrigo S. Galhardo:** conceptualization, formal analysis, funding acquisition, supervision, writing – original draft, writing – review and editing.

## Funding

This work was supported by the Fundação de Amparo à Pesquisa do Estado de São Paulo (2017/22430‐8, 2019/19435‐3, 2021/10577‐0, 2018/15819‐9, 2020/12744‐8, 2024/03230‐1, 2022/06555‐3), the Coordenação de Aperfeiçoamento de Pessoal de Nível Superior (Finance Code 01) and the Conselho Nacional de Desenvolvimento Científico e Tecnológico (305143/2022‐0).

## Conflicts of Interest

The authors declare no conflicts of interest.

## Supporting information


**Figure S1:** Growth of PAO1 and SDPAO1 strains and their derivatives after UV irradiation. Serial dilutions of cultures were spotted on LB agar plates. Plates were irradiated or not with UV‐C light as indicated and subsequently incubated in the dark at 37°C for 24 h before photographs were taken.

**Data S1:** Supplementary File.

## Data Availability

Supplemental files contain all data related to the Figures and Tables of the manuscript. Sequencing raw data are available at https://www.ncbi.nlm.nih.gov/sra/PRJNA884718.
