# Supplementary figures and images for: Roles of Alp and Prt Regulons in the Response of Pseudomonas aeruginosa to UV‐C Light
Source: Environ Microbiol Rep. 2026 Jan 12;18(1):e70268. doi: 10.1111/1758-2229.70268 (PMC12796719; doi:10.1111/1758-2229.70268)

Figure S1

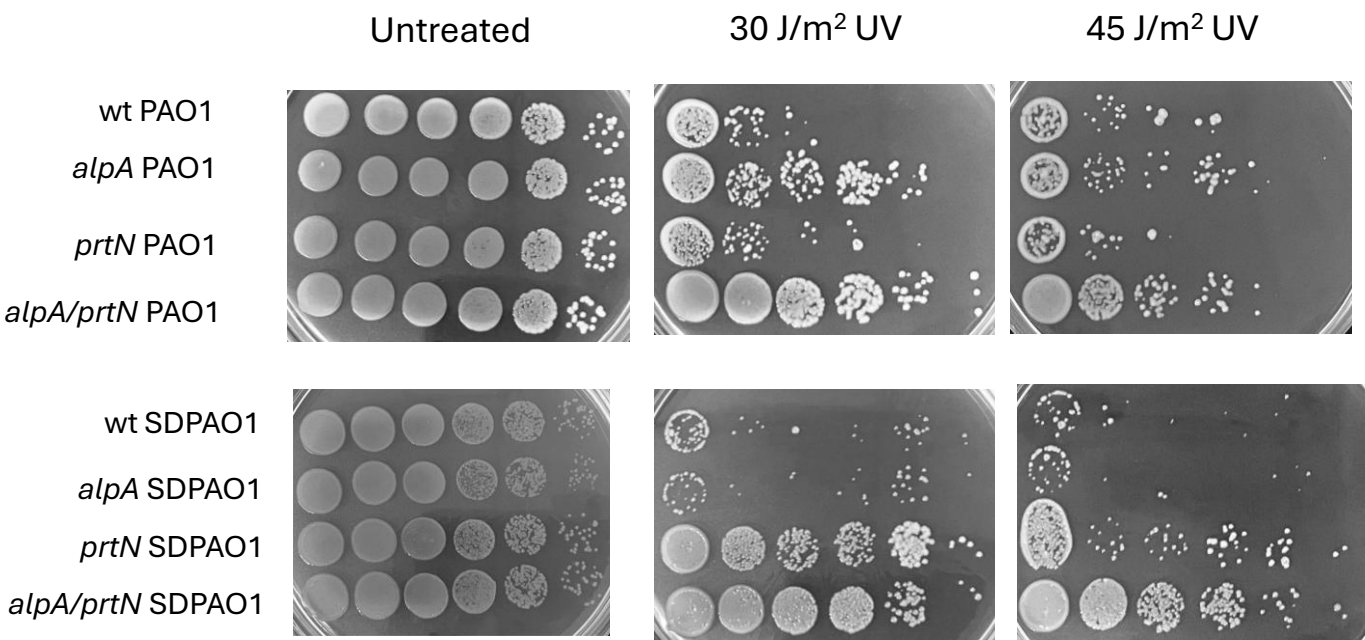

Supplement: Supplementary file 1 — Figure S1: Growth of PAO1 and SDPAO1 strains and their derivatives after UV irradiation. Serial dilutions of cultures were spotted on LB agar plates. Plates were irradiated or not with UV‐C light as indicated and subsequently incubated in the dark at 37°C for 24 h before photographs were taken. [file EMI4-18-e70268-s001.pdf]
